# Impact of Spencer Technique on Pain, Range of Motion, and Functional Disability in Patients With Frozen Shoulder: A Pilot Study

**DOI:** 10.7759/cureus.53263

**Published:** 2024-01-30

**Authors:** Pratik Phansopkar, Moh'd Irshad Qureshi

**Affiliations:** 1 Musculoskeletal Physiotherapy, Ravi Nair Physiotherapy College, Datta Meghe Institute of Higher Education and Research, Wardha, IND; 2 Neuro-Physiotherapy, Ravi Nair Physiotherapy College, Datta Meghe Institute of Higher Education and Research, Wardha, IND

**Keywords:** manual therapy technique, mobilization, spadi, frozen shoulder, spencer technique, stiffness, pain, physical therapy, codman’s exercise

## Abstract

Background and objective

The shoulder is the most flexible ball- and socket-type joint in the human body. The pathological condition that can commonly affect this joint is the frozen shoulder. This condition is marked by pain and stiffness in the area surrounding the shoulder complex. This leads to difficulty in doing the daily activities of living. Exercise and physical therapy are mostly recommended to decrease pain and improve and maintain the range of motion (ROM). Mainly traditional techniques such as Mulligans, Maitland, and Kaltenborn are used, along with electrotherapy and exercises, for the treatment of this condition. The effect of the Spencer technique is seen in baseball players' shoulder function. Thus, the purpose of this study is to determine how the Spencer approach affects patients with frozen shoulders in terms of pain, ROM, and functional impairment.

Methodology

This study included 20 patients aged between 40 and 60 years with stage 2 and 3 diagnosed frozen shoulder. This is a single-group pilot study that received the Spencer technique along with a moist heat pack and Codman's exercises on the affected shoulder for three weeks. Outcome measures used for assessment before and after treatment were the visual analog scale (VAS), shoulder ROM, and shoulder pain and disability index (SPADI). After the second, third, and sixth months, a follow-up was conducted. Two patients were lost to follow-up; consequently, statistical analysis was performed on the data from 18 patients.

Results

The current study's results suggested that there was an improvement in the mean values of VAS, ROM, and SPADI at post-three weeks, and a sustained effect was observed at the second, third, and sixth months. A statistically significant difference (*P *< 0.05) was found.

Conclusions

The study's conclusions demonstrated improved pain, ROM, and SPADI scores post-intervention. Treatment effects persisted, as seen by follow-up at the two, three, and six-month marks. As a result, the Spencer technique utilized in this pilot study on frozen shoulder patients proved effective. Also, the outcome effects were sustained, which suggests its utility in frozen shoulder rehabilitation.

## Introduction

In 1934, Codman coined the phrase *frozen shoulder*, characterizing the condition as "difficult to define, difficult to treat, and difficult to explain" [1]. Frozen shoulder (FS) has been categorized into primary and secondary conditions [2]. Whereas secondary FS is linked to a specific event, such as a recognized intrinsic or extrinsic etiology. Primary FS is characterized by an idiopathic origin that presents slowly [3]. The term "secondary systemic frozen shoulder" refers to FS that has links to medical diseases, including diabetes and thyroid issues [4].

According to descriptions, an FS is a painful ailment that causes stiffness and makes it impossible to sleep on the affected side. The pain and limitation of motion related to this illness can range from minor to severe; those who have it find it difficult to perform regular activities, including dressing, taking care of themselves, moving overhead, and getting enough sleep. It affects 2% to 5% of adults overall and 10% to 15% of those who have diabetes. The majority of those affected are female and between the ages of 35 and 65 years [5,6]. It's said that the process is self-limiting. Nonetheless, the illness may last for much longer than a year in certain cases [7,8].

Structures, including the joint capsule and subacromial bursa, have been linked to the pathophysiology of FS [9]. With advancements in arthroscopy and microbiological procedures, the rotator interval, the long head of the biceps, and the coracohumeral ligament have been linked to the pathophysiology of the disorder [10]. The related tissue has inflammatory indicators, according to modern histological investigations [11]. It has also been determined that several cytokines, including interleukin (IL)-6, tumor necrosis factor-α (TNF-α), and IL-1α and β, are present [12].

FS is divided into three consecutive stages lasting approximately 24 months in total [13]. The three stages of symptoms are freezing (lasts for three to nine months), frozen (lasts for 9-14 months), and thawing (lasts for 15-24 months) [14]. Stage 1, often referred to as the *freezing phase*, is characterized by a gradual decrease in range of motion (ROM) and persistent pain. Abduction, internal rotation, external rotation, and forward flexion are all severely limited. The loss of mobility in stage 2 is attributed to painful synovitis and a reduction in capsular volume. It has been determined that the utilization of exercise therapy plays a crucial role in managing the symptoms of FS. Recommendations for various physiotherapy modalities in FS range from electrotherapy (transcutaneous electrical nerve stimulation [TENS] and interferential therapy [IFT]) to stretching, strengthening, and manual therapy techniques. The use of joint mobilization as a manual treatment technique is increasing due to data demonstrating its ability to reduce pain and improve deficits in joint ROM [15].

To preserve and restore ROM, physical rehabilitation and exercise are often highly recommended. Patients who appear with adhesive capsulitis at stage 2 or above react best to physical therapy, stretching, and other rehab programs. The objective of stage 2 treatments is to reduce pain and inflammation, as well as capsular constriction, to reduce mobility loss. The goal of physical therapy for individuals with stage 3 FS is to increase ROM with vigorous stretching to treat substantial loss of motion [16].

The Spencer approach is a widely used set of standardized shoulder treatments that can be applied to diagnosis, prognosis, and treatment. The mobilization of the scapulothoracic and glenohumeral joints is the main goal of this well-known osteopathic manipulative treatment. It enhances the function of the limited joints and has a favorable impact on other cognitive, social, and emotional domains [17-20]. The seven different treatments of the Spencer approach are used to treat adhesive capsulitis-related shoulder limitation. The purpose of this technique is to stretch constricted muscles, ligaments, and capsules using passive, smooth, rhythmic movements. In the final ROM, the majority of the force is applied. This method stimulates greater joint circulation, improves lymphatic flow, and stretches the tissues to promote ROM without pain [21].

## Materials and methods

A pilot study was carried out at the musculoskeletal outpatient department of the Acharya Vinoba Bhave Rural Hospital and Ravi Nair Physiotherapy College, Sawangi, Meghe, Wardha, India. The study obtained permission from the Institutional Ethical Committee of Datta Meghe Institute of Higher Education and Research (DU) (Ref. No. DMIMS (DU)/IEC/2021/634) and was registered with the Clinical Trial Registry of India (CTRI/2022/01/039290). The study only included participants who were permitted to participate. Twenty participants were included in the study. Outcome measures were evaluated at baseline, posttreatment at the end of the third week, and subsequently at the second, third, and sixth months. Two patients were lost to follow-up, and a statistical analysis was conducted on data from the remaining 18 patients.

Inclusion and exclusion criteria

**Figure 1:**
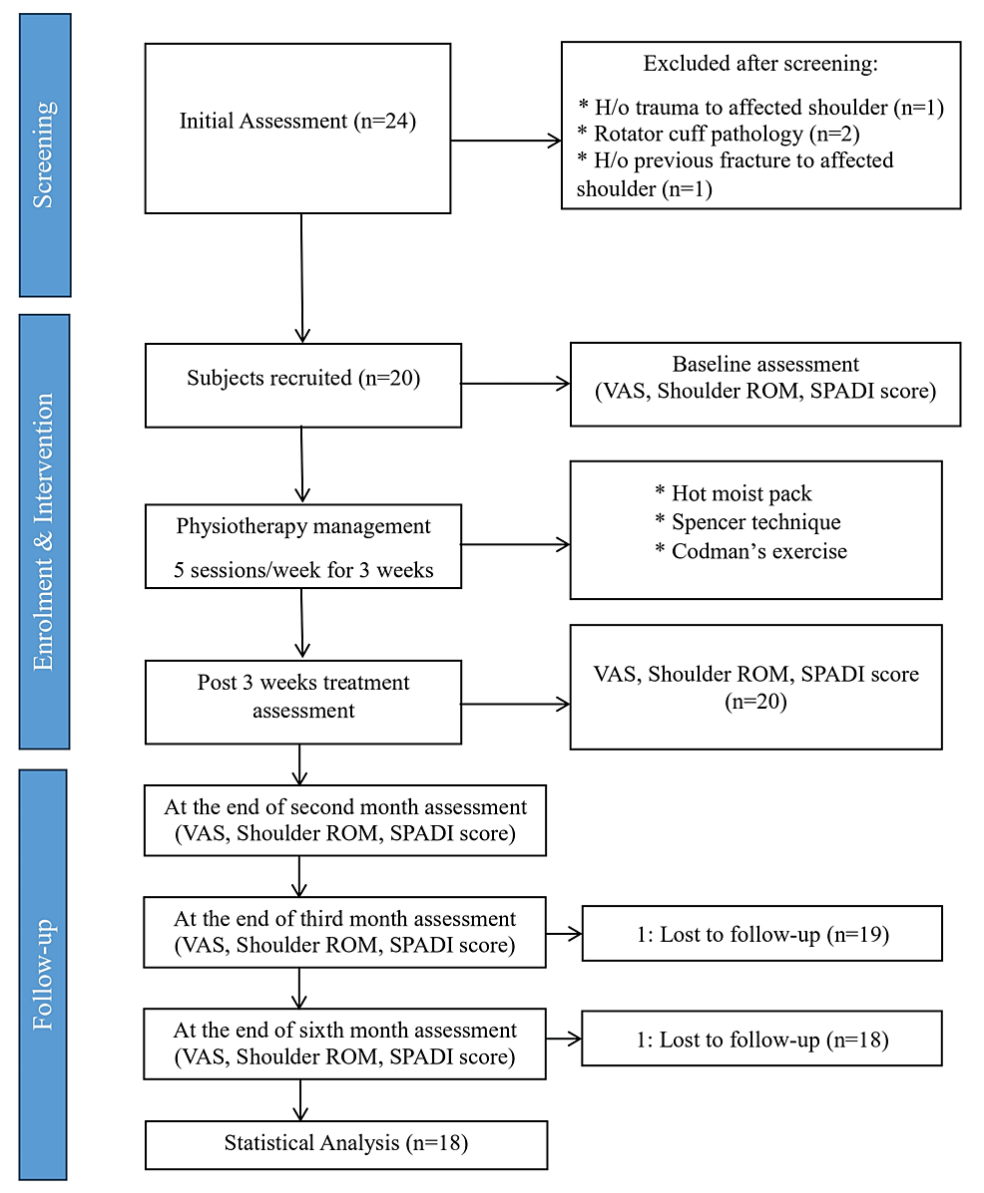
Flowchart of the sample enrollment process, follow-up, and analysis. Figure credit: Pratik Phansopkar VAS, visual analog scale; ROM, range of motion; SPADI, Shoulder Pain and Disability Index

Patients aged 40 to 60 with diabetes, regardless of gender, diagnosed with stage 2 or 3 FS (characterized by unilaterally painful and stiff shoulder for at least three months), capable of understanding commands, and willing to participate in the study were enrolled. Exclusions from the study included patients with a recent history of surgery on a specific shoulder, post-traumatic shoulder discomfort, and stiffness, a history of fracture at the shoulder complex, rib fracture, rotator cuff pathology, and tendon calcification. Figure 1 shows the methodology of the study.

Procedure

**Figure 2:**
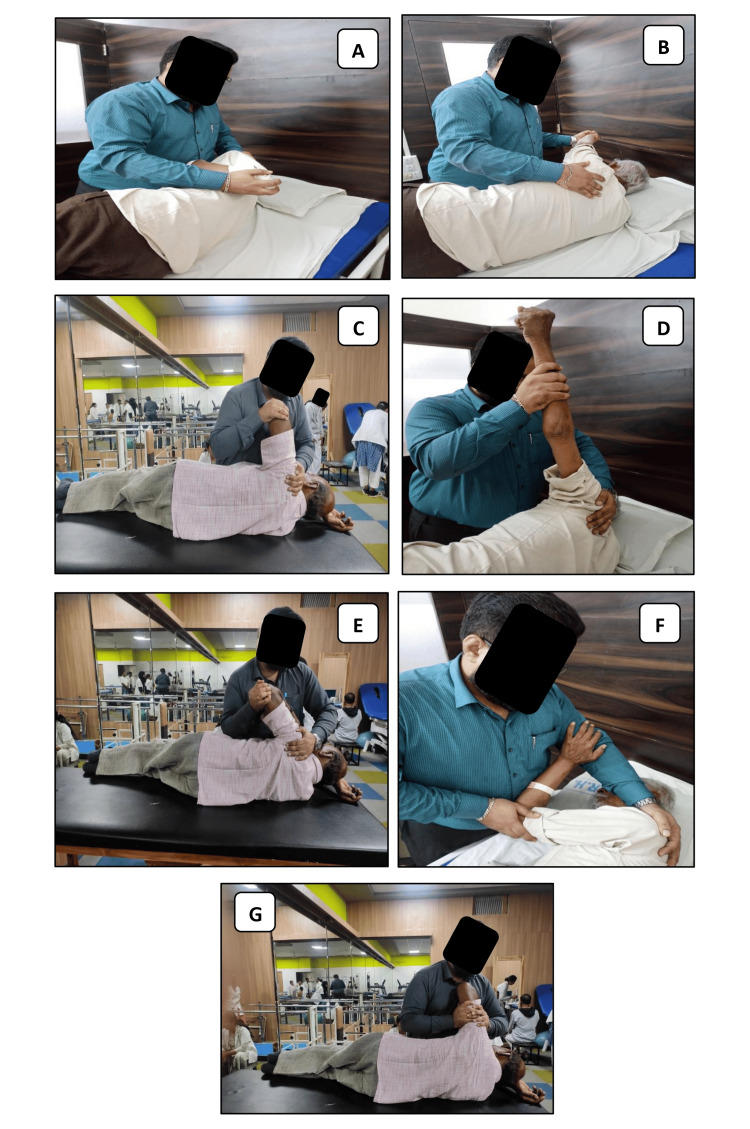
Application of the Spencer technique on the left shoulder. (A)-(G): Steps 1 to step 7 of the Spencer technique application.

The included patients received a moist hot pack over the affected shoulder for 20 minutes, after which the Spencer technique was performed, as shown in Figure 2.

Step 1: Shoulder extension with elbow flexion: The patient's elbow was maintained in a flexed position, and the arm was extended until the restricted barrier.

Step 2: Shoulder flexion with elbow extension: The patient's flexed elbow was extended and moved anteriorly into shoulder flexion until the restricted barrier.

Step 3: Circumduction with compression: Grasped the patient's elbow with the shoulder in a 90° abduction, and moved the elbow in small clockwise and counterclockwise circles with a compressive force.

Step 4: Circumduction with distraction: The therapist maintained the traction of the patient's shoulder joint in 90° of abduction and held either elbow or wrist-induced small clockwise and counterclockwise circles.

Step 5: Shoulder abduction and internal rotation with elbow flexion: The patient was asked to place his hand on the therapist's forearm for support, and then the therapist performed abduction and internal rotation of the patient's arm. Internal rotation (90°) - The therapist placed the dorsum of the patient's hand behind his or her hip and moved the patient's elbow anteriorly.

Step 6: Shoulder adduction and external rotation with elbow flexion: The patient was asked to place his hand on the therapist's forearm for support, and then the therapist took the patient's arm into adduction and external rotation.

Step 7: Stretching tissue and pumping fluids with the arm extended: The therapist interlocked his fingertips over the deltoid muscle, the patient‘s hand was placed over the therapist's shoulder, and the therapist slowly moved the arm away from the shoulder and released, repeated this 5-10 times.

For five sessions each week, it was administered for two sets of 10 repetitions with a one-minute break in between, increasing to three sets of 10 repetitions in the second and third weeks.

Patients were instructed to undertake the Codman's exercise, which comprised standing with the affected arm hanging down and the trunk flexed from the waist with an uninvolved hand supporting them. The patient was also instructed to rock their body in a circular motion without tensing their shoulder muscles. The arm was moved in both clockwise and anticlockwise directions, as well as side to side and back and forth. In the first week, it was given for one set for five sessions/week and increased to two sets for five sessions/week in the second and third weeks. The implementation of a home exercise program involving Codman's exercises was recommended, and an exercise chart was provided for guidance.

Visual analog scale (VAS), shoulder flexion, extension, abduction, internal and external rotation ranges, and Shoulder Pain and Disability Index (SPADI) score were assessed pre- and posttreatment as outcome measures.

Statistical analysis

For the statistical analysis, *P *< 0.05 was used as the significance level. Students paired t-test was used for descriptive and inferential statistics using IBM SPSS Statistics for Windows, Version 27.0 (IBM Corp., Armonk, NY) software and GraphPad Prism 7.0 (GraphPad Software, San Diego, CA).

## Results

Twenty patients were enrolled in the study, and each participant's eligibility was determined. Out of this, one patient lost follow-up at the end of the third month due to lack of transportation and one patient at the end of the sixth month due to relocation to a different town, respectively. So, the data analysis of 18 patients was done at the end of the six-month follow-up to see the sustainable effect of the treatment. The findings of the current study suggest an improvement in the mean values of VAS, ROM, and SPADI at post-three weeks, with a sustained effect observed at the second, third, and sixth months. A statistically significant difference was noted (*P *< 0.05). Tables 1-2 display the mean differences of the variables used as outcome measures posttreatment.

**Table 1:** Mean differences of VAS and SPADI scores after three weeks posttreatment and follow-up at the second, third, and sixth months. ^*^Significant. Pre indicates the baseline score. VAS interpretation: 0-4 mm (no pain), 5-44 mm (mild pain), 45-74 mm (moderate pain), and 75-100 mm (severe pain). VAS, visual analog scale; SPADI, Shoulder Pain and Disability Index (a score of 0 indicates best, 100 indicates worst)

Variable (I)	Variable (J)	Mean difference (I - J) VAS	*P*-value	Mean difference (I - J) SPADI	*P*-value
Pre	Third week	1.95^*^	<0.05	26.65^*^	<0.05
	Second month	3.45^*^		39.15^*^	
	Third month	4.80^*^		44.45^*^	
	Sixth month	4.80^*^		44.45^*^	
Third week	Pre	-1.95^*^	<0.05	-26.65^*^	<0.05
	Second month	1.50^*^		12.50^*^	
	Third month	2.85^*^		17.80^*^	
	Sixth month	2.85^*^		17.80^*^	
Second month	Pre	-3.45^*^	<0.05	-39.15^*^	<0.05
	Third week	-1.50^*^		-12.50^*^	
	Third month	1.35^*^		5.30^*^	
	Sixth month	1.35^*^		5.30^*^	
Third month	Pre	-4.80^*^	<0.05	-44.45^*^	<0.05
	Third week	-2.85^*^		-17.80^*^	
	Second month	-1.35^*^		-5.30^*^	
	Sixth month	0.000	1.000	0.000	1.000
Sixth month	Pre	-4.80^*^	<0.05	-44.45^*^	<0.05
	Third week	-2.85^*^		-17.80^*^	
	Second month	-1.35^*^		-5.30^*^	
	Third month	0.000	1.000	0.000	1.000

**Table 2:** Mean difference in the range of motion of the shoulder posttreatment at the end of the third week and follow-up conducted at the end of the second, third, and sixth months. ^*^Significant. Pre indicates the baseline score. The unit of ROM is degrees (°). Normal shoulder ROM: flexion (0°-180°), extension (0°-60°), abduction (0°-180°), internal rotation (0°-70°), external rotation (0°-90°). ROM, range of motion

Variable (I)	Variable (J)	Mean difference (I - J) flexion ROM	*P-*value	Mean difference (I - J) extension ROM	*P-*value	Mean difference (I - J) abduction ROM	*P*-value	Mean difference (I - J) internal rotation	*P-*value	Mean difference (I - J) external rotation	*P*-value
Pre	Third week	-54.2^*^	<0.05	-7.75^*^	<0.05	-40.95^*^	<0.05	-216^*^	<0.05	-22.75^*^	<0.05
	Second month	-55.55^*^	<0.05	-9.45^*^	<0.05	-40.95^*^	<0.05	-25.95^*^	<0.05	-25.15^*^	<0.05
	Third month	-55.55^*^	<0.05	-9.45^*^	<0.05	-44.90^*^	<0.05	-25.95^*^	<0.05	-25.15^*^	<0.05
	Sixth month	-55.55^*^	<0.05	-9.45^*^	<0.05	-44.90^*^	<0.05	-25.95^*^	<0.05	-25.15^*^	<0.05
Third​​​​​​​ week	Pre	54.2^*^	<0.05	7.75^*^	<0.05	40.95^*^	<0.05	21.65^*^	<0.05	22.75^*^	<0.05
	Second month	-1.35	0.883	-1.70	0.493	0.000	1.000	-4.30^*^	<0.05	-2.40^*^	0.017
	Third​​​​​​​ month	-1.35	0.883	-1.70	0.493	-3.95	0.669	-4.30^*^	<0.05	-2.40^*^	0.017
	Sixth​​​​​​​ month	-1.35	0.883	-1.70	0.493	-3.95	0.669	-4.30^*^	<0.05	-2.40^*^	0.017
Second​​​​​​​ month	Pre	55.55^*^	<0.05	9.45^*^	<0.05	40.95^*^	<0.05	25.95^*^	<0.05	25.15^*^	<0.05
	Third​​​​​​​ week	1.35	0.883	1.70	0.493	.000	1.000	4.30^*^	<0.05	2.40^*^	0.017
	Third​​​​​​​ month	0.000	1.000	0.000	1.000	-3.95	0.669	0.000	1.000	0.000	1.000
	Sixth​​​​​​​ month	0.000	1.000	0.000	1.000	-3.95	0.669	0.000	1.000	0.000	1.000
Third​​​​​​​ month	Pre	55.55^*^	<0.05	9.45^*^	<0.05	44.90^*^	<0.05	25.95	<0.05	25.15	<0.05
	Third​​​​​​​ week	1.35	.883	1.70	.493	3.95	.669	4.30	<0.05	2.40	0.017
	Second​​​​​​​ month	0.000	1.000	0.000	1.000	3.95	0.669	0.000	1.000	0.000	1.000
	Sixth​​​​​​​ month	0.000	1.000	0.000	1.000	0.000	1.000	0.000	1.000	0.000	1.000
Sixth​​​​​​​ month	Pre	55.55^*^	<0.05	9.45^*^	<0.05	44.90^*^	<0.05	25.95	<0.05	25.15	<0.05
	Third​​​​​​​ week	1.35	0.883	1.70	0.493	3.95	0.669	4.30	<0.05	2.40	0.017
	Second​​​​​​​ month	0.000	1.000	0.000	1.000	3.95	0.669	0.000	1.000	0.000	1.000
	Third​​​​​​​ month	0.000	1.000	0.000	1.000	0.000	1.000	0.000	1.000	0.000	1.000

## Discussion

This study was designed to evaluate the effects of the Spencer technique on ROM, functional impairment, and pain among patients with stage 2 or stage 3 FS. The time intervals for assessing outcomes post-intervention were set to monitor the sustainability of improvements, as there are chances of reversals of impairments. The outcome demonstrated a notable posttreatment improvement, and the impact persisted during multiple follow-up visits. The pain was reduced, and the range improved, indicating an improvement. The outcome measures - VAS, shoulder ROM, and SPADI - showed improvement in the post-intervention assessment and were sustained in all follow-up assessments at different time intervals. Reduced pain and improved shoulder movement facilitated the positive impact on the patient's shoulder function, which has been demonstrated in this study with a reduced SPADI score post-intervention and also in follow-up assessments. Initially, the Spencer technique was used in the rehabilitation of a patient with an FS, and the patient experienced favorable outcomes as a result of undergoing rehabilitation using the Spencer technique, which prompted the researchers to undertake a pilot study to investigate further and potentially validate the effectiveness of this technique in a broader context [22].

It has been demonstrated that stretching and the application of moist heat enhance muscular extensibility. This could happen as a result of neuromuscular-mediated relaxation and a decrease in muscle viscosity [23].According to a study by Chan et al., stretching and the application of moist heat increase muscular extensibility [24].

Spencer's method restores specific joint motion by stretching the shoulder capsule and taut soft tissues, increasing the amount of pain-free ROM. When used, this method improves the lymphatic flow out of the treated area. This method resets neural reflexes and restores the joint's normal ROM. Improved lubrication, nourishment, and circulation in the joint structures are achieved through passive repetitive translation movements, traction, or gliding. It restores the joint's detrimental alterations and returns arthokinematic rolling and gliding to normal. Greater gliding will allow for the restoration of shoulder mobility by restoring osteokinematic rotation to normal [7].

Additionally, tissue alterations, pain, asymmetry, and restricted motion - physical markers of somatic dysfunction - are diminished through the Spencer approach [18]. The basic mechanism for pain relief is altered by this manipulation method, which also changes the amounts of circulatory pain biomarkers. Soft tissue stretching and fluid mobilization are two additional physiological mechanisms that contribute to the efficacy of the Spencer technique, enhancing glenohumeral and scapulothoracic joint mobility [18,19]. To improve shoulder complex mobility, it addresses the motions that cause the least amount of pain first, followed by the most restriction. With the Spencer approach, low-threshold mechanoreceptors in muscles and joints are stimulated. This causes sympatho-excitation stimulation to be produced by the somatic efferent neurons, which helps to locate activation in the midbrain's periaqueductal gray matter. Nociceptive inhibitors from the midbrain inhibit nociceptive impulses in the spinal cord's dorsal horn by shutting the gate [18-20]. Consequently, this pain gate pathway's mechanoreceptors in muscles and joints are activated to control or suppress pain [25].

With the help of Codman Pendular Exercise, soft tissue components like muscles and tendons can be stretched, maintaining their flexibility and increasing ROM in the shoulder joint. This will naturally boost functional activity and reduce pain. A similar benefit of Codman's exercise in cases with FSs was demonstrated by Selviani et al. in their study [26]. Owing to this physiological impact, improvements were seen in the VAS, shoulder ROM, and SPADI scores following treatment.

The lack of ongoing follow-up of two patients and unsupervised adherence to the home program unfavorably affected the comprehensive assessment and tracking of outcomes, introducing a potential limitation to the reliability and completeness of the study findings. This study also involved a smaller sample size as it was a pilot study, and further studies could compare this intervention protocol with a control group.

## Conclusions

The study's findings demonstrated that the Spencer technique is effective in reducing pain, improving shoulder ROM, and decreasing functional impairment in patients with FSs. This study also elicited the improvements being sustained throughout the time interval follow-up that were assessed; hence, the Spencer technique should be considered in treating patients with FSs. Further research is recommended with a larger sample size and compared with other available manual therapy methods.
